# Study of Single and Multipass *f*–rGO Inkjet-Printed Structures with Various Concentrations: Electrical and Thermal Evaluation

**DOI:** 10.3390/s23042058

**Published:** 2023-02-11

**Authors:** Apostolos Apostolakis, Dimitris Barmpakos, Aggelos Pilatis, Vassiliki Belessi, Dimitrios-Nikolaos Pagonis, Fadi Jaber, Konstantinos Aidinis, Grigoris Kaltsas

**Affiliations:** 1 microSENSES Laboratory, Department of Electrical and Electronics Engineering, University of West Attica, 12244 Athens, Greece; 2 Department of Naval Architecture, University of West Attica, 12244 Athens, Greece; 3 Department of Graphic Design and Visual Communication, Graphic Arts Technology Study Direction, University of West Attica, 12243 Athens, Greece; 4 Department of Biomedical Engineering, Ajman University, Ajman P.O. Box 346, United Arab Emirates; 5 Center of Medical and Bio-Allied Health Sciences Research, Ajman University, Ajman P.O. Box 346, United Arab Emirates; 6 Department of Electrical and Computer Engineering, Ajman University, Ajman P.O. Box 346, United Arab Emirates

**Keywords:** graphene, reduce graphene oxide (rGO), inkjet-printing technique, flexible substrates, electrical characterisation, thermal characterisation

## Abstract

Reduced graphene oxide (rGO) is a derivative of graphene, which has been widely used as the conductive pigment of many water-based inks and is recognized as one of the most promising graphene-based materials for large-scale and low-cost production processes. In this work, we evaluate a custom functionalised reduced graphene oxide ink (*f*–rGO) via inkjet-printing technology. Test line structures were designed and fabricated by the inkjet printing process using the *f*–rGO ink on a pretreated polyimide substrate. For the electrical characterisation of these devices, two-point (2P) and four-point (4P) probe measurements were implemented. The results showed a major effect of the number of printed passes on the resulting resistance for all ink concentrations in both 2P and 4P cases. Interesting results can be extracted by comparing the obtained multipass resistance values that results to similar effective concentration with less passes. These measurements can provide the ground to grasp the variation in resistance values due to the different ink concentrations, and printing passes and can provide a useful guide in achieving specific resistance values with adequate precision. Accompanying topography measurements have been conducted with white-light interferometry. Furthermore, thermal characterisation was carried out to evaluate the operation of the devices as temperature sensors and heaters. It has been found that ink concentration and printing passes directly influence the performance of both the temperature sensors and heaters.

## 1. Introduction

Inkjet-printing, a noncontact printing technique, attracts scientific interest as it is an enabler technology in the field of flexible electronics and is already utilized in a wide range of applications [1,2]. It is an efficient way to deposit functional materials on either flexible or rigid substrates in order to form a desired pattern. A pattern in a digital format is directly deposited on the substrate without the use of masks. The ink is dropped at a precisely defined point on the substrate, creating the designed printing patterns [3]. Additionally, no special processing conditions are needed and several inks with different characteristics could be used effectively with minor modifications, depending on the printer’s nozzle, the printed pattern, the ink type, the application of the device, etc., in order to develop several predefined printing patterns in flexible substrates, even in the nanometer range [4]. The requirement for different types of applications of printable electronics require the development of inks and substrates with different property sets and characteristics. As the complexity of the designed structures increase, the required materials’ complexity also increases. Inkjet-Printing Technology (IPT), compared to other printing methods, presents compatibility with a wide variety of inks and substrates, such as plastic, paper, textile, etc. [5,6,7], with low material consumption and almost zero waste [8]. It is effective for the fabrication of a variety of complex electronic components and devices such as: sensors (gas [9,10], temperature [11,12,13,14], and humidity [14,15]), microheaters [16,17,18], energy harvesters, capacitors, FETs, etc. [19,20,21,22].

Typically, most flexible printed electronic applications are based on organic or polymer inks, due to their characteristics such as conductivity, electrical and mechanical stability, and compatibility with a variety of substrates [23,24], while maintaining biocompatibility [25,26,27]. Nevertheless, it is important to measure and accurately define the optimal conditions for printing conductive lines using inkjet; there have been various efforts towards accurately measuring the resistivity of inkjet-printed structures [28,29], mainly because they are used for interconnections [30,31], electrode fabrication [32], etc. The replicability of printed structures is vital for actually defining the large-scale production capabilities of the material technology stack, with failures affecting device integrity [33], performance [34,35], and accurate fabrication [36].

Heating small areas with microheaters is an important task for several applications, from flow-sensing [37,38], degassing, driving and assisting electrochemical sensors [39,40,41,42,43,44], to microfluidic system temperature control and sensing [45,46,47]. Carbon and graphene-based printed devices have recently demonstrated unprecedented performance in temperature sensing and microheater fabrication [16,48,49,50,51]; due to their unique properties, such as high mechanical durability, resistance to environmental corrosion and contamination, and an ability to reach high temperatures without changing their properties, these materials are excellent candidates for these applications. Functionalised reduced graphene oxide has also recently been utilized as a heater material [52].

In this work, we extend the preliminary study conducted for accurate and repeatable patterning by the inkjet-printing of a custom *f*–rGO ink [48]; detailed electrical characterisation has been performed as a function of printing passes and concentration, followed by electro-thermal characterisation. Moreover, several surface topography measurements have been implemented in order to examine further the impact of the different printing passes and concentrations to the samples’ roughness. A correlation between the printing parameters and the extracted resistivity was experimentally extracted. In this way, the optimal parameter set can be defined for each application and especially for microheater fabrication.

## 2. Materials and Methods

### 2.1. Materials

Graphite (powder, synthetic, particle size < 20 μm), potassium chlorate (purum > 99.0%) and 2,4—diaminobenzenesulfonic acid (98%) were purchased from Merck, KGaA, (Darmstadt, Germany). Νitric acid (65%) and sulfuric acid (95–97%) were purchased from Riedel-de Haen (Munich, Germany) and Merck, KGaA, (Darmstadt, Germany), respectively, and were used as provided.

#### 2.1.1. Synthesis of Graphite Oxide

Graphite oxide was prepared by a modified Staudenmaier method [53]. Specifically, powdered graphite was added (2 g) under stirring to a mixture consisting of nitric acid (40 mL) and sulfuric acid (80 mL). The reaction took place in a Pyrex flask immersed in an ice-bath. Then, potassium chlorate (40 g) was slowly added while maintaining the cooling and stirring conditions. The reactions were quenched after 18 h by pouring the mixture into deionized water. The product was washed thoroughly with deionized water until pH ~ 6 and dried at room temperature.

#### 2.1.2. Synthesis of *f*–rGO

Graphite oxide (200 mg) was dispersed in deionized water (200 mL) and remained for 24 h under stirring. After that, the aqueous graphene oxide dispersion (1 mg/mL) was sonicated for 30 min in a Branson 3800 bath sonicator (110 W, 40 kHz). Then, 600 mg of 2,4—diaminobenzenesulfonic acid (2,4—DBSA) was added to the GO dispersion; the mixture was refluxed under magnetic stirring for 2 h and, after cooling, was vacuum filtered using Nylon membrane filters (0.45 μm pore size, Whatman). The product (*f*–rGO) [54,55] was washed extensively with deionized water, ethanol, and acetone. For this work, an appropriate amount of the air-dried *f*–rGO was dispersed in deionized water in order to obtain three different *f*–rGO ink concentrations, namely: 2.5 wt%, 5 wt%, and 10 wt%, and all devices were printed using these concentration values.

### 2.2. Design and Fabrication of Printed Devices

A Drop on Demand (DOD) semicustom inkjet-printer (Thetametrisis FR—DEPOSIT) equipped with a Microdrop MD-K-140 nozzle (diameter: 70 μm) and MD-6020 head controller was used for the fabrication process. The setup is equipped with a USB3 camera (MQ013MG-E2, XIMEA) to monitor droplet formation using stroboscopic imaging. A typical test line structure was designed and fabricated by the inkjet-printing process using a custom functionalised reduced graphene oxide *f*–rGO ink on a polyimide substrate (DuPont Kapton HN, Wilmington, DE, USA; thickness 125 μm). The substrate was pretreated with 1M NaOH for 8 min, for increasing wettability; prior to printing, the substrate has been washed in an ultrasonic bath with deionized water, isopropyl alcohol, and acetone [56,57].

**Figure 1:**
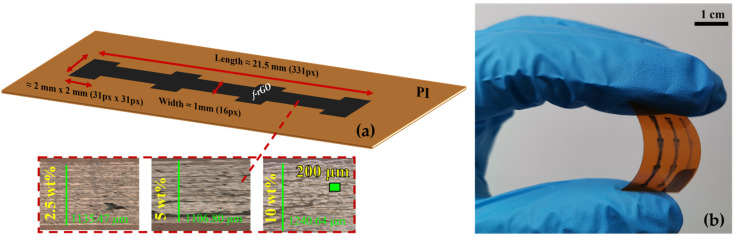
(**a**) Test line structure geometries and top view details from optical microscope for various ink concentrations; (**b**) fabricated devices with a single and multipass approach.

More specifically, the test line was designed with a length of 21.5 mm (331 px) and a width of 1 mm (16 px); additionally, four rectangular pads were designed with the same geometries of 2 mm × 2 mm (31 px × 31 px) at equal distances from each other. This device was formed in both a single and multipass approach. In the second approach, one/two/three-printing pass was performed with a droplet spacing of 65 μm in both axes. To calculate the actual dimensions of the printed devices, we consider that the diameter of each droplet is 65 μm and corresponds to one printed pixel. It has been found experimentally that the optimal morphology for *f*–rGO is achieved in the case where there is no overlap between successive droplets. Therefore, the distance of consecutive pixels is considered 65 μm and the final dimension of the printed structure is calculated from the relationship: length = (px number) × 65 μm. Figure 1a presents the dimension parameters of the designed geometry and the corresponding images, which were extracted from the optical microscope, and Figure 1b depicts the fabricated devices with a single and multipass approach. It should be noted that the actual samples’ size may vary slightly due to deviations in the droplets during the inkjet-printing process. In addition to multipass evaluation, a concentration-related study was also conducted. Therefore, all lines were inkjet-printed (using 2.5 wt%, 5 wt%, and 10 wt% *f*–rGO concentrations) and subsequently placed in an oven for 1 h at 85–90 °C. The sintering process and the curing conditions for the custom *f*–rGO ink were determined in a previous work [48]. Before the printing process, each *f*–rGO ink concentration was sonicated for 3 min at 35 Watt. All the samples were placed on to the printer’s integrated hotplate, which was set up at room temperature (25 °C), which ensures the repeatability of the process and contributes positively to the degassing of the substrate, in order to avoid any effect from environmental humidity.

The optimal printing settings for all three concentrations were experimentally extracted by fine-tuning all the related parameters. A droplet spacing of 65 μm in both axes resulted in good droplet overlap, while a piezoelectric driving pulse of −80 V for 40 μs (for 2.5 wt% and 10 wt%) and −80 V for 60 μs (for 5 wt%), result in stable unified droplets from the nozzle. Table 1 summarises the optimal droplet setting, as well as all the printing parameters for each *f*–rGO ink concentration.

**Figure 2:**
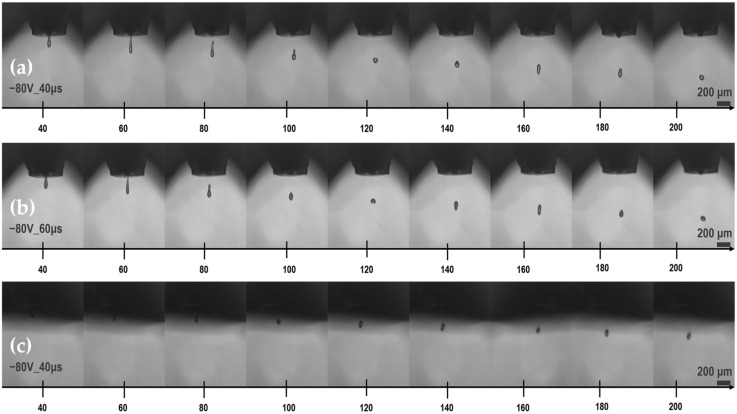
Droplet formation of *f*–rGO ink: (**a**) 2.5 wt% (−80 V/40 μs); (**b**) 5 wt% (−80 V/60 μs); (**c**) 10 wt% (−80 V/40 μs).

Figure 2 presents the selected piezoelectric settings at 20 μs steps, starting from 40 μs after first firing.

### 2.3. Electrical Characterisation—Surface Topography Study

**Figure 3:**
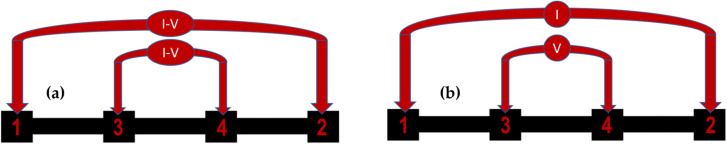
Measurement setup electrical characterisation: (**a**) two-point configuration; (**b**) four-point configuration.

The printed samples were characterised using a typical probe station connected to a Keithley 2612 source meter with a custom LabVIEW interface, alongside a needle prober. More specifically, in order to electrically characterise these devices, a two-point and a four-point probe measurement was implemented. For all samples, the same I—V curves were acquired using the two-point measurement technique in two different points, as is shown in Figure 3a. Furthermore, for the four-point technique, resistance was determined by applying a current to points 1 and 2, and simultaneously measuring the potential difference between points 3 and 4, as shown in Figure 3b.

To extract the resistivity value for each printed device, the thickness (t) was experimentally determined using a Filmetrics Profilm3D optical profiler (Unterhaching, Germany). The Profilm3D optical profiler uses state of the art white-light interferometry (WLI) to measure surface profiles and roughness [58] and is located on top of a tabletop active antivibration stage (DVIA-T45, Daeil Systems) in order to isolate the signals from environmental vibrations. WLI exploits the difference in distance travelled by the light from a sample to an objective lens detector, where the light from the source is separated into reference and measurement beams. The measurement beam is then used to extract information regarding surface roughness and topology [59]. To extract the thickness (t) of each sample, an automated step-height analysis offered by the related software was used.

Finally, using the optical profiler described above, an area roughness analysis has also been implemented. The inspected area was 250 μm × 180 μm (width × height) at all samples and no levelling, flattening or filtering techniques have been previously applied. Roughness parameters were defined automatically by the following standards: ASME B46.1 3D, EUR 15178N amplitude and ISO 25178 height.

### 2.4. Thermal Characterisation Setup

The thermal-electrical characterisation of the devices was performed using two different techniques, based on the external and internal heating of the devices.

**Figure 4:**
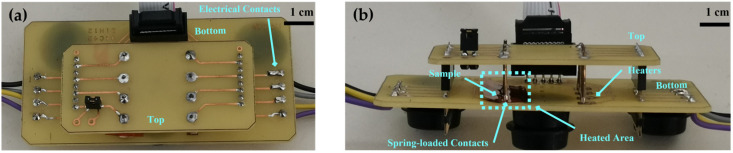
External thermal measuring experimental setup using Pt100 microheaters: (**a**) top view; (**b**) cross-section.

Initially, in the external heating case, a custom PCB circuit with standard Pt100 heating elements was used, that was driven by a constant current from a Keithley 2612 source meter. The substrate temperature was continuously monitored by a high-precision FLIR A655SC IR camera. Current–voltage measurements were performed through a typical probe station in the temperature range between 35 and 65 °C. All measurements have been taken at points 1 and 2. Details on the custom experimental setup have already been described in a previous study [48], and are presented in Figure 4. In summary, at the top of the PCB, two rows of spring-loaded contacts were used in parallel as engagement and measuring units. The bottom layer consisted of two rows of commercial Pt100 SMT thermistors, which were used as heating elements. An IDC10 connector served as an interface with external instruments to drive the SMT heaters; the devices under test could be positioned vertically or horizontally, depending on the dimensions and the experimental requirements.

**Figure 5:**
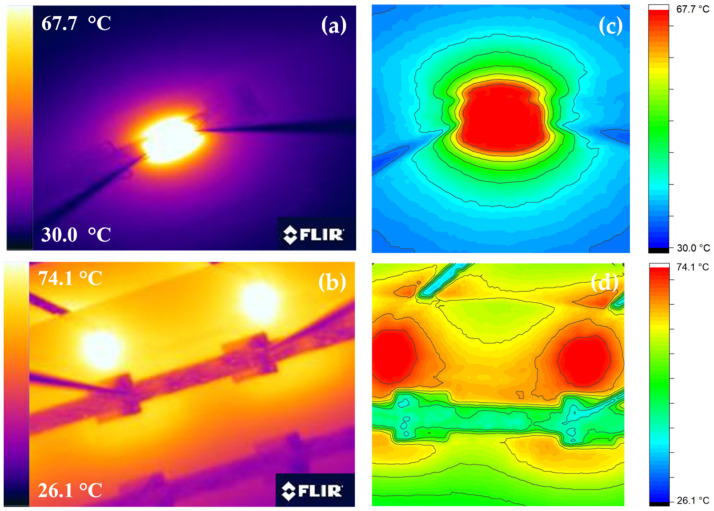
IR images and thermal contour plots (**a**,**c**) for self-heated case; (**b**,**d**) external heating implementation. Hot spots created by the external heaters are visible, but uniform on-sample temperature distribution is achieved.

Following the first characterisation, the internal heating thermal evaluation was performed in order to assess the operation of the printed devices as heaters. Six distinct current pulses were applied and the corresponding voltage was measured. In all cases, the IR camera was used to monitor the temperature response to different power inputs. Figure 5 depicts the IR and contour plots of the thermal images for both implemented methods for typical heating scenarios. Isothermal contours reveal a uniform heating profile around the sample, while it should be noted that all the self-heating temperature profiles exhibit the same pattern. When external heating is applied underneath the samples, we can observe that although hot spots are evident over the SMT elements, the sample reaches a relatively uniform temperature.

## 3. Results and Discussion

### 3.1. Resistance Study

**Figure 6:**
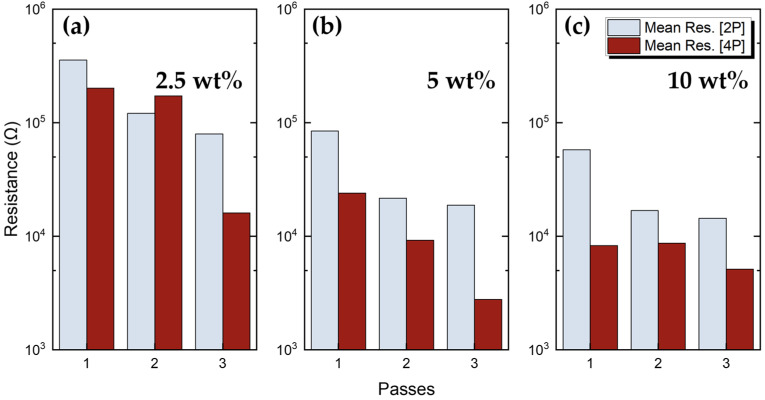
Two-point (2p) and four-point (4p) resistance measurements, as a function of different printing passes for: (**a**) 2.5 wt%; (**b**) 5 wt%; (**c**) 10 wt% *f*–rGO ink concentrations.

All printed devices were electrically characterised using the experimental setup described in Section 2.2. In agreement with similar works [48,60,61], the results (Figure 6) showed a significant effect of the number of printed passes on the resulting resistance for all ink concentrations in both 2P and 4P cases. All ink concentrations exhibited a variance of less than 10%. Resistance dropped almost exponentially with the number of passes in all concentration values (with one exception for 10 wt% concentration); thus, a higher drop is observed between the one-pass and two-pass cases. A difference in the resistance values between 2P and 4P measurements is indicated in Figure 6, which may have occurred due to the probe location engagement; however, the trend is similar.

**Figure 7:**
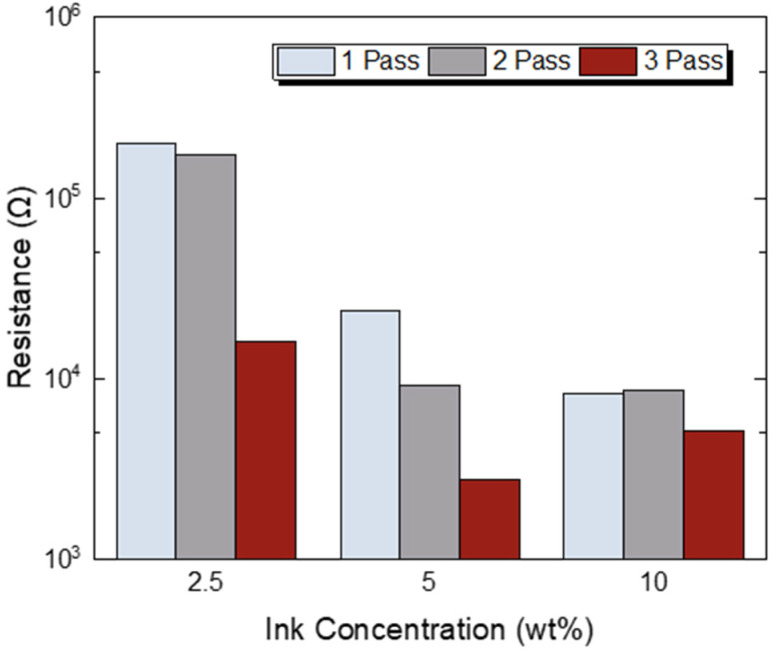
Four-point (4p) resistance as a function of *f*–rGO ink concentrations for different printing passes.

Figure 7 presents the resistance as a function of *f*–rGO ink concentrations for different printing passes. Interesting results can be extracted by comparing the obtained multipass resistance that results in similar effective concentration with less passes (e.g., two-pass 5 wt% and one-pass 10 wt% resistance value). In all cases, a major drop in the resistance occurs at three-passes, while the resistance value is almost constant in one- and two-passes for both 2.5 wt% and 10 wt% cases. Consequently, these measurements can provide the ground to grasp the variation in resistance values due to the different ink concentrations and printing passes, and can provide a useful guide in achieving specific resistance values with adequate precision.

Except from the typical resistance evaluation, it is of particular interest to investigate the mean resistivity in each case. To fabricate stable and reproducible inkjet-printed devices the accurate determination of the resistivity is a crucial parameter.

### 3.2. Resistivity Extraction

**Figure 8:**
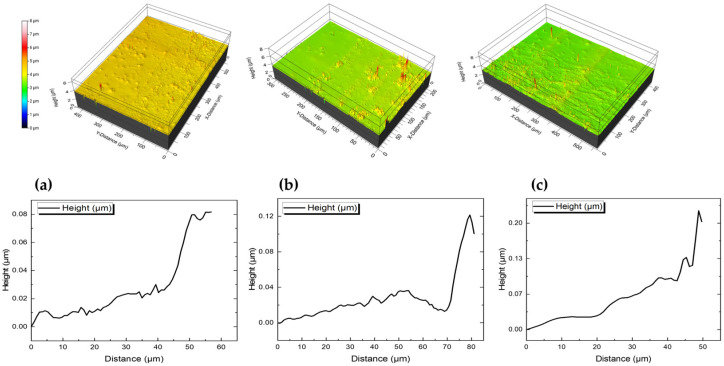
3D height optical profile measurements for: (**a**) 2.5 wt%; (**b**) 5 wt%; (**c**) 10 wt%; *f*–rGO ink concentrations (top view and height profiles).

In order to calculate the resistivity of each sample, the actual thickness value should be determined. The measurement of the thickness of an inkjet-printed sample is not a trivial process, especially in overprinted structures. To extract the absolute height of each device, an optical profiler setup was used as already described in detail in Section 2.2. Figure 8 depicts the 3D measurement results for three samples printed with three different ink concentrations (three-pass cases).

To evaluate the mean resistivity of the printed samples, the standard resistivity definition formula [62] was applied, according to Equation (1):

ρ = R × A/L(1)

where A (width × thickness) is the cross-sectional area, R is the measured resistance using the four-point technique, and L is the distance between the probes (3, 4). Table 2 summarises the results of the resistivity values for the *f*–rGO printed samples, along with the thickness of each printed layer that was used for the evaluation using the four-point measurement technique. The dimensions of all samples are 5.4 mm × 1 mm.

**Figure 9:**
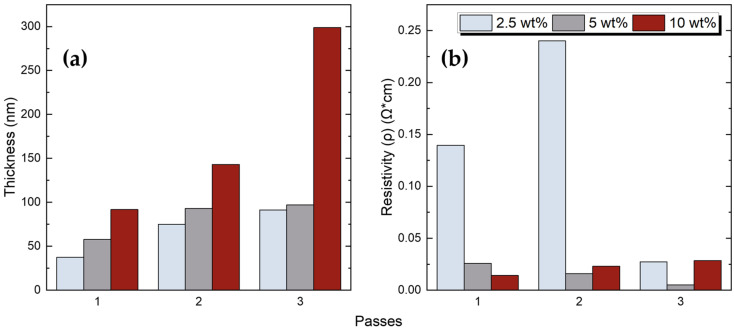
(**a**) Thickness of each printed device; (**b**) resistivity values as a function of printing passes for different *f*–rGO concentrations.

For the same number of passes, the layer thickness increases with ink concentration, as expected (Figure 9a). The thickness increase is more pronounced between two- and three-passes, especially for the 10 wt% concentration. Accordingly, a thickness increase is observed with the increase of the number of passes in a specific concentration. Similarly, the effect is enhanced between two- and three-passes. The resistivity behaviour is not following the trend indicating in Figure 7. For the 2.5 wt% concentration, a peak is observed for the two-pass case, while a smooth decreasing behaviour is extracted for the 5 wt% case. On the contrary, a relatively small increase is indicated for the 10 wt% case, mainly between a single and two-pass case (Figure 9b). For the single pass samples, the resistivity drops almost exponentially with the concentration; however, when more passes are implemented then more factors contribute to the final resistivity value, such as the interface between the ink layers, the type and the amount of the ink mixing, and the potential insulating intermediate barrier layers.

**Figure 10:**
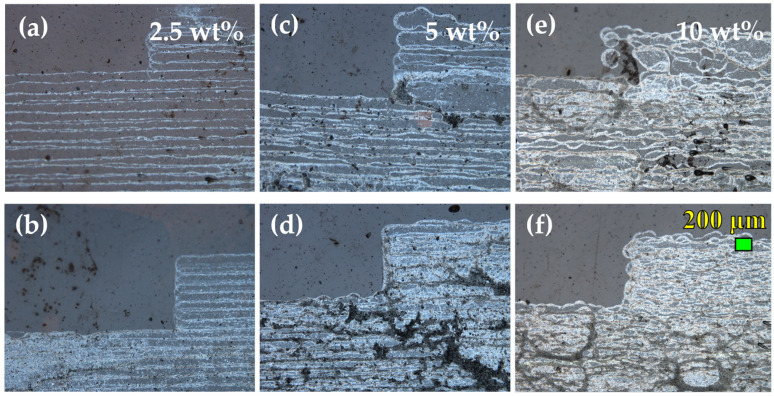
Optical microscope images for different *f*–rGO concentrations: (**a**,**c**,**f**) one-pass; (**b**,**d**,**e**) three-pass pads of the devices.

Figure 10 presents optical microscopy images of all the samples under evaluation. At 2.5 wt% concentration, although relatively repeatable lines have been achieved, a low quantity of the active material results in failure to form a uniform material layer, even in three passes of printing. In both 5 wt% and 10 wt% cases, it is evident that a higher number of passes result in continuous material layers with varying roughness areas. Additionally, material agglomerations can be observed in overprinted samples. The simultaneous reduction and surface functionalisation of GO by diamino benzene sulfonic acid led to highly dispersible and stable graphene derivatives [54,55]. The as-prepared functionalised rGO is highly water dispersible, without the need of surfactants or other additives in order to fine-tune the viscosity and surface tension and make the dispersions compatible with inkjet-printing. Moreover, such an ink formulation ensures compatibility with various substrates, since the curing process temperature remains low. However, the use of water as the sole solvent can cause, in some cases, the formation of black spots in inkjet-printed samples, for several reasons. For example, the duration of the inkjet-printing process is several minutes (up to 45 min for three passes) per sample. During this period the *f*–rGO dispersion remains in a vessel since the inkjet-printer machine implements the continuous flow principle. This can cause possible agglomerates when printed in multiple passes. Furthermore, the low viscosity of the water leads to deviations in the droplets during the inkjet-printing process. Therefore, it is important to find the optimal printing conditions (e.g., ink concentration, printing passes, curing time and temperature, substrate treatment properties, etc.), in order to minimise this issue.

### 3.3. Surface Topography Measurements

In order to perform a consistent roughness analysis, several roughness parameters were extracted using the experimental setup described in Section 2.2. Surface roughness is the calculation of the relative smoothness of a surface profile from its ideal form. Although many parameters are required to describe effectively the actual surface topology, in this work we indicatively present the two most commonly used parameters. Sa is the arithmetic average of the peaks and valleys of the device surface, including the profile height deviations of the mean line. This variable is the most commonly used metric; the higher the deviations, the rougher the surface, thus small Sa values indicate smooth surfaces. Sq is the root mean square of the surface roughness. These two measurement parameters could be considered as a consistent evaluation factor for surface smoothness because of their precision in the measurement process, and their availability as reference values on common profilometers. Table 3 presents Sa and Sq values for all *f*–rGO printed samples.

**Figure 11:**
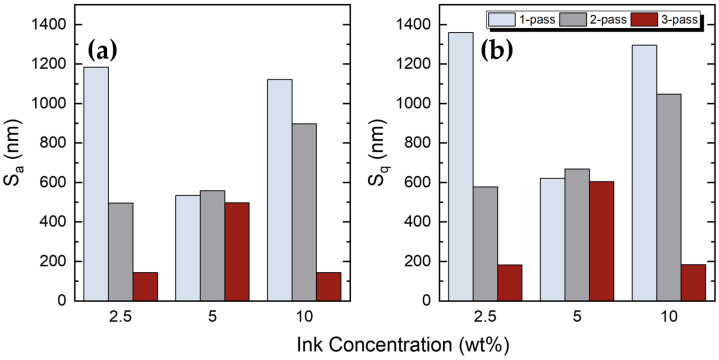
(**a**) Arithmetic mean height (Sa) between the roughness profile and the mean line; (**b**) root mean square of the surface roughness (Sq) as a function of different printing passes and ink concentrations.

Figure 11 depicts the Sa and Sq values as a function of different printing passes and ink concentrations.

Both Sa and Sq parameters present similar behaviour in all the cases. In 2.5 wt% and 10 wt% concentrations, the roughness decreases with the number of printing passes and a major drop is observed between the two- and three-pass case. On the contrary, the surface roughness remains almost the same at 5 wt% concentration. The roughness behaviour in relation to the number of passes varies with the concentration, thus in the 5 wt% concentration, the single pass samples indicate a minimum, while the three-pass samples present a maximum roughness. An increasing behaviour with concentration is observed for two-pass samples. The best results in terms of consistency are extracted in the 5 wt% case, where the surface roughness is almost constant with the number of passes, which indicates an enhanced repeatability of the corresponding printed structures.

### 3.4. Thermal Evaluation

#### 3.4.1. External Thermal Evaluation

**Figure 12:**
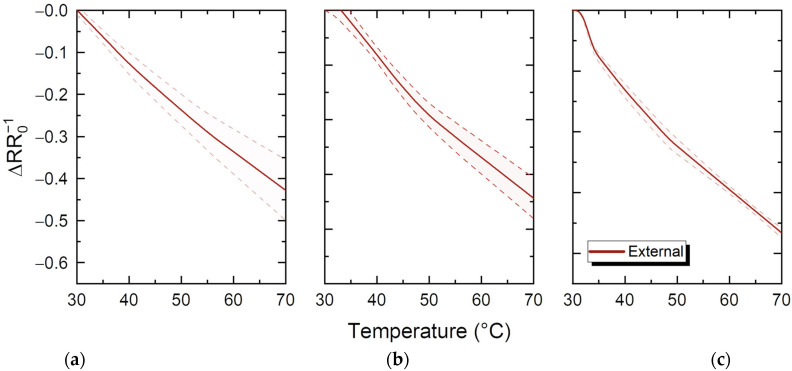
Normalised temperature–resistance relationship for the *f*–rGO printed samples of (**a**) 2.5 wt%; (**b**) 5 wt%; (**c**) 10 wt% (average of all printing passes with standard deviation band) for external thermal evaluation.

All samples have been thermally evaluated in the range from 30 to 70 °C. For each concentration, mean resistance and standard deviation bands have been extracted for single, double, and triple printed samples, and are depicted in Figure 12. Higher concentrations seem to have narrower standard deviation band, i.e., the number of passes does not have a big impact on the temperature coefficient of resistance; in lower concentrations, additional printing passes influence the TCR more, while in 10 wt%, the TCR is similar for all printing passes. This can be attributed to the fact that in lower concentrations, the amount of material for single and double pass is actually not enough to reach the TCR of the bulk material; this can also explain the slightly higher response achieved by higher concentration.

#### 3.4.2. Internal Thermal Evaluation (Self-Heating)

**Figure 13:**
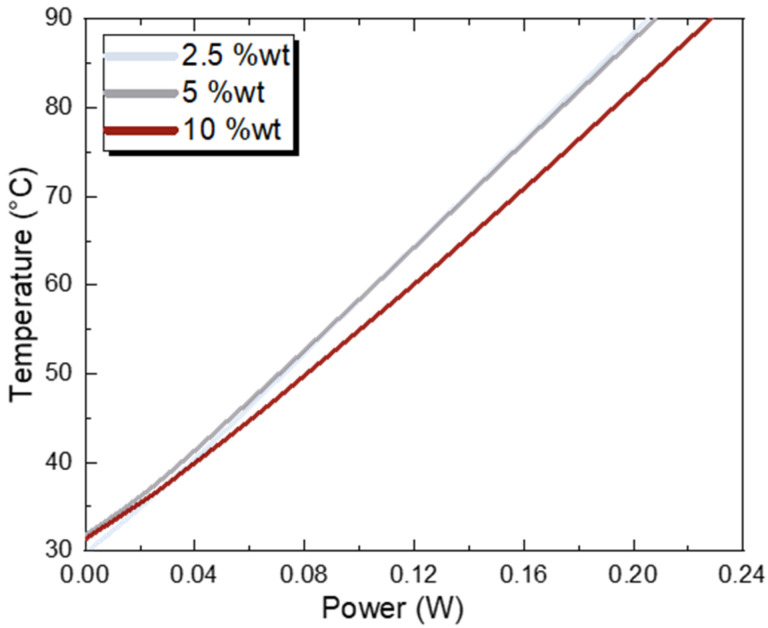
Temperature–power relationship for the *f*–rGO printed samples as a function of the different ink concentrations (average of all printing passes).

The specific material can operate either as sensing or as active heating material. For such applications, the device efficiency is determined by the amount of applied power in order for the heater to reach a specific temperature. The less power needed, the more efficient the device is. In all of the experiments, the setup was placed inside a closed fume hood in order to maintain controlled environmental conditions so as to avoid external disturbances and flow over the samples. As seen in Figure 13, the 2.5 wt% and 5 wt% samples exhibit identical behaviour; 90 °C was reached by providing 208 mW to the samples. For the same temperature setpoint, samples with 10 wt% required 228 mW, approximately 4.5% more. In combination with the morphological studies, one can explain this additional power requirement by the fact that the 10 wt% samples have larger profiles; that is, more thermal mass to heat with the same thermal coefficient of resistance.

## 4. Conclusions

In this work, the multipass inkjet-printed structures of three concentrations of *f*–rGO water-based ink were evaluated for their electrical, morphological, and thermal properties on Kapton substrate.

All *f*–rGO ink concentrations were capable of creating continuous, electrically conductive lines on the Kapton substrate in both one-pass or multipass approaches. In terms of resistance, all samples exhibit a variance of less than 10%. Additionally, resistance dropped with the number of printing passes at all ink concentrations. Furthermore, line thickness, and consecutive resistivity, were found to vary with concentrations, with 5 wt% exhibiting the most stable behaviour in terms of interpass resistivity difference.

For extracting thermal-electrical characteristics, two sets of experiments were conducted. The samples were initially heated externally with platinum microheaters and it was found that 10 wt% material had a lower standard deviation between samples, with a higher TCR. This can be attributed to the fact that more dense printed material is closer to the materials’ bulk TCR. Self-heating experiments were also carried out in the range from 30 to 90 °C. It was found that higher-concentration required more power to reach the same temperature, a result that is consistent with the thickness analysis, which presented thicker layers for higher-concentration samples. The specific material can operate either as sensing or as active heating material. For such applications, the device efficiency is determined by the amount of applied power.

Additionally, surface topography measurements have been implemented, which show that roughness is directly dependent on the printing passes. Further, the more consistent results were extracted again in the 5 wt% case, which indicates an enhanced repeatability of the printing processes.

Finally, the optical images revealed defects in the printing quality of the samples (black spots–agglomerated rGO) especially in overprinted structures. This could be mainly attributed to the fact that *f*–rGO is a water-based ink. This innovative feature enables its compatibility with various substrates, though it may sometimes compromise the printing quality. The printing quality of the samples can be impacted by multiple factors (e.g., ink concentration, printing passes, curing time and temperature, substrate treatment properties, etc.), leaving room for further investigation into the optimal printing conditions for this innovative material.

This study could contribute to the effective exploitation of this material in a wide variety of applications using inkjet-printing or a combination of different printing technologies. It could also be a useful guide to optimize printing processes and lead to more repeatable results. Future steps include the further study of this innovative material and its composites, especially in terms of the fabrication of more complex electronic elements such as diodes, capacitors, and FETs, etc.

## Figures and Tables

**Table 1:** Printing properties classified by ink concentration.

Settings	2.5 wt%/10 wt%	5 wt%
Droplet spacing (x,y) (μm)	65, 65	65, 65
Voltage (V)	−80	−80
Pulse Duration (μs)	40	60

**Table 2:** The summarised results of the resistivity values for the *f*–rGO printed samples.

Concentration	Passes	Resistance (kΩ)	Thickness (t) (nm)	Resistivity (ρ) (Ω cm)
2.5 wt%	1	201.00	37.30	0.13948
	2	172.00	75.00	0.24000
	3	16.10	91.26	0.02734
5 wt%	1	24.00	57.76	0.02581
	2	9.20	92.98	0.01591
	3	2.78	97.06	0.00502
10 wt%	1	8.30	91.65	0.01415
	2	8.70	141.30	0.04836
	3	5.14	298.90	0.02858

**Table 3:** The summarised results of the area roughness analysis for the *f*–rGO printed samples.

Concentration	Passes	Arithmetic Mean Height, Sa (nm)	Root Mean Square Height, Sq (nm)
2.5 wt%	1	1184	1359
	2	496	577
	3	144	182
5 wt%	1	534	620
	2	558	668
	3	497	604
10 wt%	1	1121	1295
	2	897	1047
	3	144	184

## Data Availability

The data presented in this article are available on request from the corresponding author.
